# Supplementary material for: Nonthermal Plasma Synthesis of Metallic Ti Nanocrystals
Source: Nanomaterials (Basel). 2024 Jan 26;14(3):264. doi: 10.3390/nano14030264 (PMC10856339; doi:10.3390/nano14030264)
Supplement: Supplementary file 1 [file nanomaterials-14-00264-s001.zip › nanomaterials-2833183-supplementary.pdf]

nanomaterials

Article

Supplementary Information

Qiaomiao Tu [1], David L. Poerschke [1,*] and Uwe R. Kortshagen [2,*]

[1] Department of Chemical Engineering and Materials Science, University of Minnesota, Minneapolis, MN 55455, USA; tu000007@umn.edu
[2] Department of Mechanical Engineering, University of Minnesota, Minneapolis, MN 55455, USA
* Correspondence: dpoersch@umn.edu (D.L.P.); korts001@umn.edu (U.R.K.)

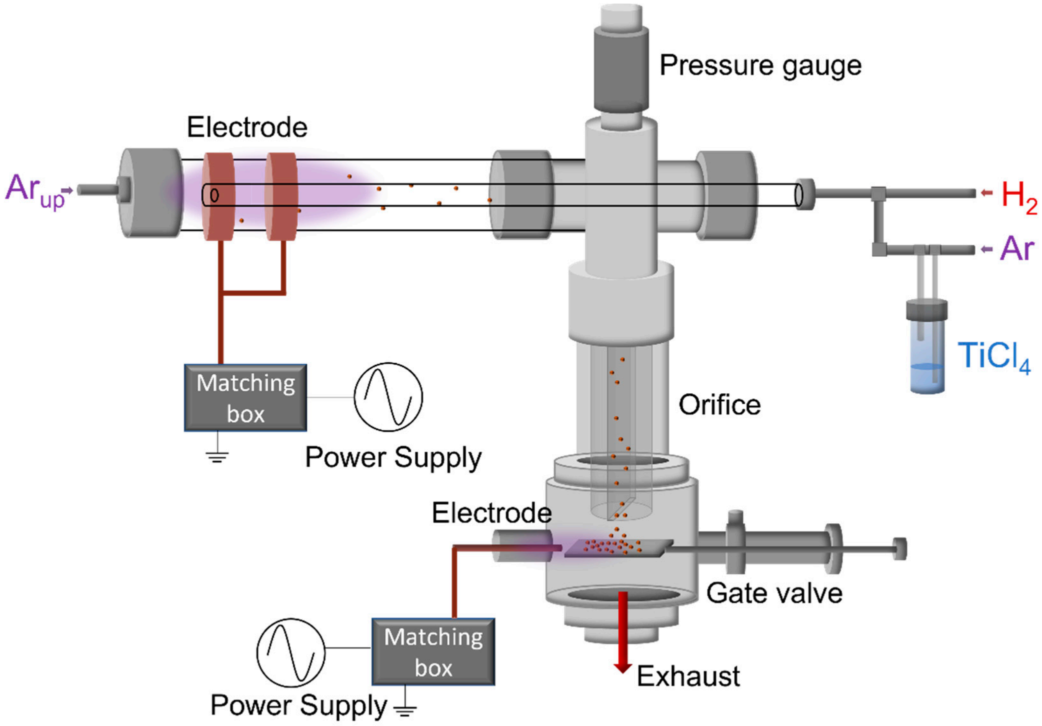

Figure S1. Schematic of the nonthermal plasma reactor for Ti nanoparticle production

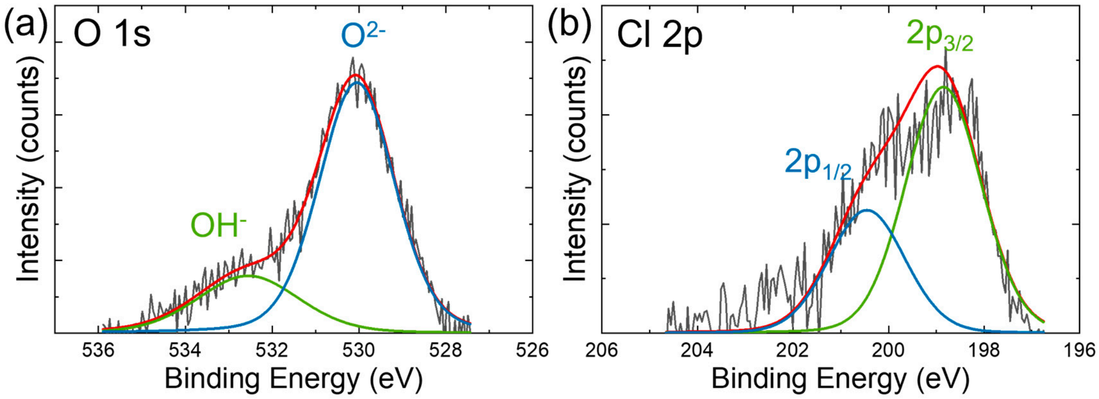

Figure S2. XPS high-resolution spectra of (**a**) O 2p and (**b**) Cl 2p of as-synthesized nanoparticles as seen in Figure 1

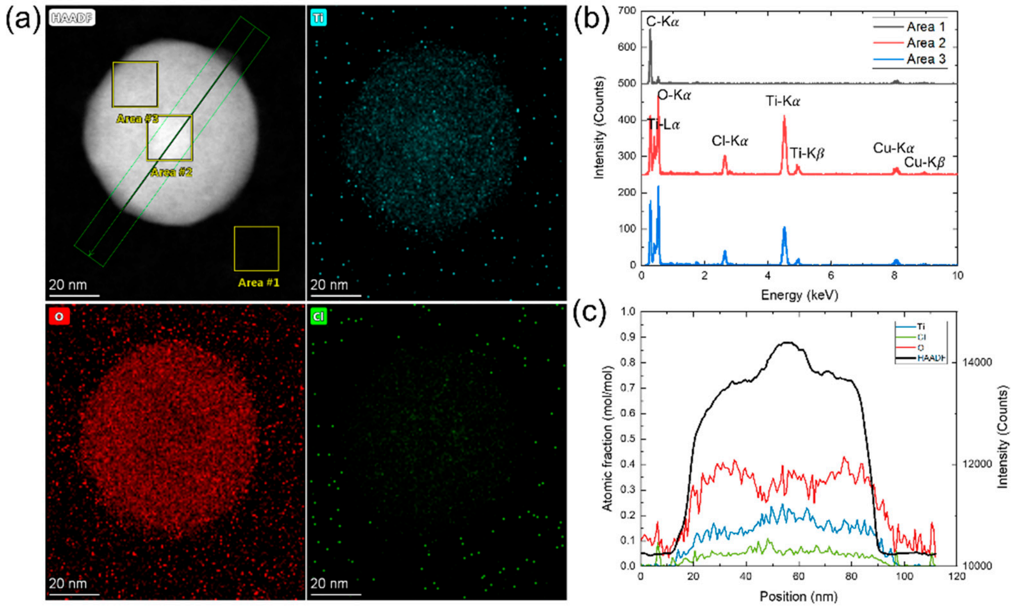

Figure S3. HAADF-STEM-EDS mapping images of as-synthesized nanoparticles as seen in Figure 1. (**a**) HAADF and EDS mapping images (**b**) EDS of three selected areas from (**a**) (**c**) line scanning profile across the particle as indicated in (**a**).

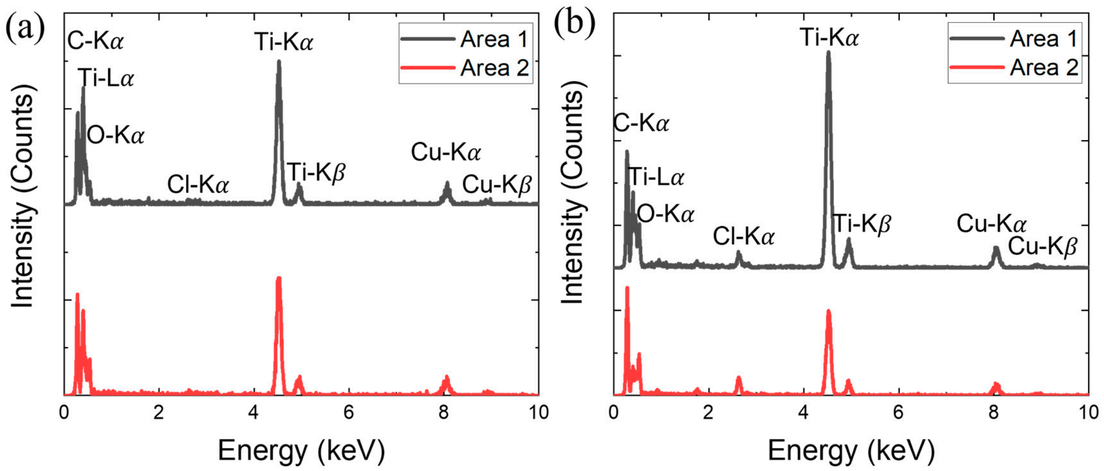

Figure S4. EDS of the (**a**) cubic nanoparticle and (**b**) hexagonal nanoparticle selected from two areas, area 1 at the particle center, area 2 at the periphery indicated in Figure 3 (**a**) and Figure 4 (**a**).

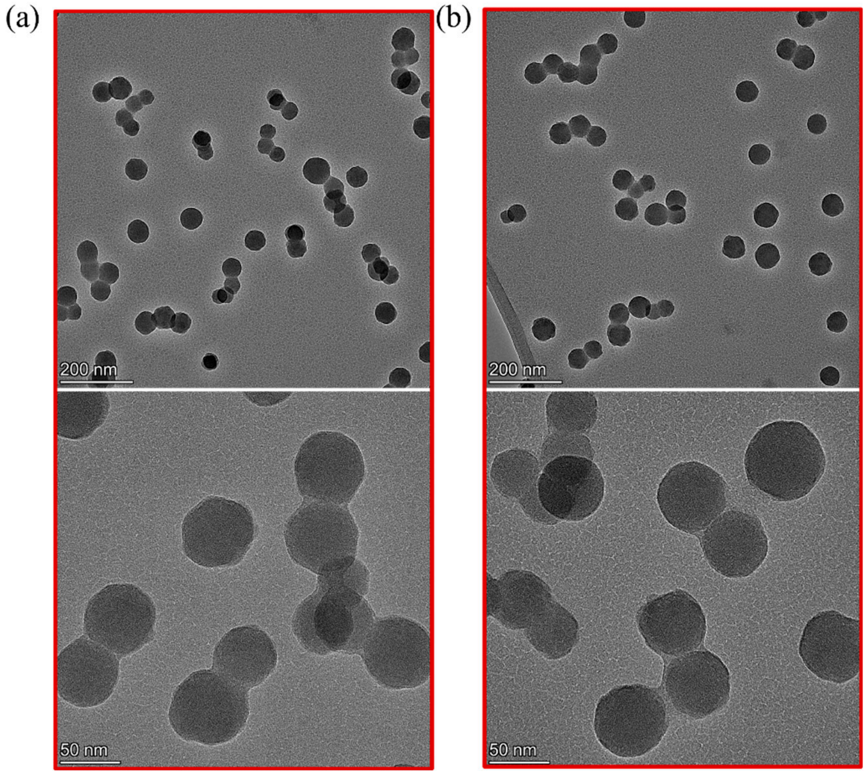

Figure S5. (**a**) CTEM images of oxidized Ti nanoparticles after exposure to the air (**b**) CTEM images of Ti nanoparticle treated with the secondary H_2 plasma after exposure to the air.

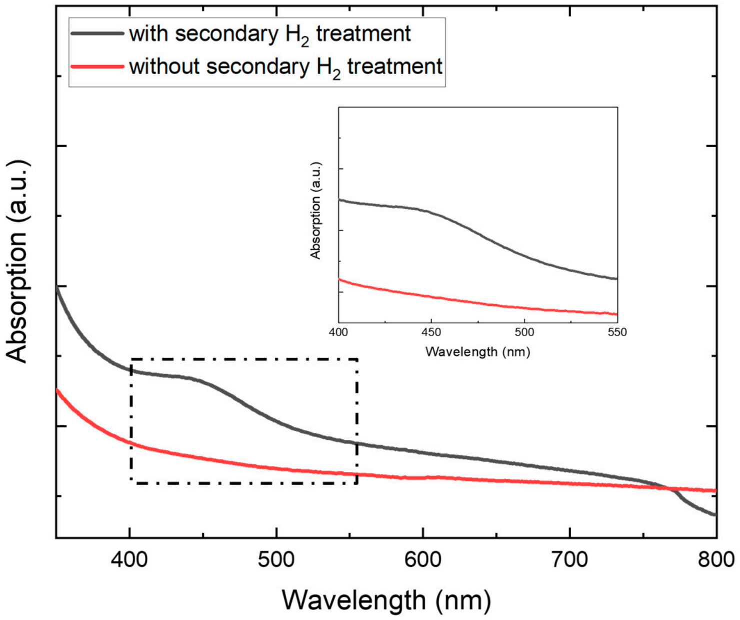

Figure S6. UV-Vis absorbance spectra of Ti nanoparticles with and without secondary H_2 plasma treatment, inset shows the zoomed in view of 400-550 nm range of the spectra.

Table S1. Plasma synthesis conditions.

Conditions	Values
Electrode	CCP
Power	50 - 150 W
Ar_{up} flow rate	25 sccm
H_2 flow rate	180 sccm
$Ar(TiCl_4)$ flow rate	15 sccm
$TiCl_4$ bubbler temperature	0 ℃
$TiCl_4$ bubbler pressure	236 Torr
Chamber pressure	2.5 Torr

Table S2. Predicted parameters from the optimized line profile fitting.

Fitted element	Hexagonal shaped particle		Cubic shaped particle	
	Parameter	Fitted values	Parameter	Fitted values
O	R_c	34 nm	R_c	27 nm
	R_s	46 nm	R_s	37 nm
	a	0.31	a	0.40
Cl	R_c	29 nm	R_c	31 nm
	R_s	46 nm	R_s	37 nm
	b	0.13	b	0.08

Line profile mode

A cubic core-shell structure was used to model the cubic nanoparticles and a spherical core-shell structure was used to approximate the hexagonal nanoparticle. 2D projections of the 3D models are shown in Figure 3 (b) and 4 (b), respectively. The core radius is denoted as R_c and the particle radius is denoted as R_s. At a certain beam path distance p from the center of the particle, the 1D EDS intensity counts along the beam axis are assumed to be proportional to two section of the beam path through the particle, the section in the core region with length L_c and the two sections in the shell region with combined length L_s.

For the cubic model, the lengths are calculated as

$$L_c = \begin{cases} 2 \times R_c & (0 \le p \le R_c) \\ 0 & (R_c < p \le R_s) \end{cases}$$

$$L_s = \begin{cases} 2 \times (R_s - R_c) & (0 \le p \le R_c) \\ 2 \times R_s & (R_c < p \le R_s) \end{cases}$$

Likewise, for the spherical model, the lengths are calculated as

$$L_c = \begin{cases} 2 \times \sqrt{{R_c}^2 - p^2} & (0 \le p \le R_c) \\ 0 & (R_c < p \le R_s) \end{cases}$$

$$L_s = \begin{cases} 2 \times (\sqrt{{R_s}^2 - p^2} - \sqrt{{R_c}^2 - p^2}) & (0 \le p \le R_c) \\ 2 \times \sqrt{{R_s}^2 - p^2} & (R_c < p \le R_s) \end{cases}$$

By assuming a core of pure metallic Ti and a shell of $Ti_{1-a-b}O_aCl_b$, the intensity counts of Ti, O, and Cl are expressed as

$$I_{Ti} = L_c + L_s \times (1 - a - b)$$

$$I_O = L_s \times a$$

$$I_{Cl} = L_s \times b$$

We specify the normalized root mean square error (NRMSE)

$$NRMSE = \frac{\sqrt{\sum(x - \hat{x})^2}}{\sqrt{\sum(x - \bar{x})^2}}$$

as the cost function and then calculate the goodness of fit. Here, x represents the experimental data, $\hat{x}$ the predicated values, and $\bar{x}$ the mean of the experimental data.
